# Effects of school-based interventions on mental health stigmatization: a systematic review

**DOI:** 10.1186/1753-2000-2-18

**Published:** 2008-07-21

**Authors:** Howard M Schachter, Alberta Girardi, Mylan Ly, Denise Lacroix, Andrew B Lumb, Judith van Berkom, Ritu Gill

**Affiliations:** 1 Provincial Centre of Excellence for Child and Youth Mental Health at CHEO, Ottawa, Ontario, Canada; 2 Department of Pediatrics, Faculty of Medicine, University of Ottawa, Ottawa, Ontario, Canada; 3 Children's Hospital of Eastern Ontario (CHEO) Research Institute, Ottawa, Ontario, Canada

## Abstract

Stigmatizing, or discriminatory, perspectives and behaviour, which target individuals on the basis of their mental health, are observed in even the youngest school children. We conducted a systematic review of the published and unpublished, scientific literature concerning the benefits and harms of school-based interventions, which were directed at students 18 years of age or younger to prevent or eliminate such stigmatization. Forty relevant studies were identified, yet only a qualitative synthesis was deemed appropriate. Five limitations within the evidence base constituted barriers to drawing conclusive inferences about the effectiveness and harms of school-based interventions: poor reporting quality, a dearth of randomized controlled trial evidence, poor methods quality for all research designs, considerable clinical heterogeneity, and inconsistent or null results. Nevertheless, certain suggestive evidence derived both from within and beyond our evidence base has allowed us to recommend the development, implementation and evaluation of a curriculum, which fosters the development of empathy and, in turn, an orientation toward social inclusion and inclusiveness. These effects may be achieved largely by bringing especially but not exclusively the youngest children into direct, structured contact with an infant, and likely only the oldest children and youth into direct contact with individuals experiencing mental health difficulties. The possible value of using educational activities, materials and contents to enhance hypothesized benefits accruing to direct contact also requires investigation. Overall, the curriculum might serve as primary prevention for some students and as secondary prevention for others.

## Background

The earliest usage of the term "stigma" referred to the act of "branding" [1], which entailed cutting or burning signs into the body to expose something unusual or bad about the moral status of the "marked" person. These marks warned others that the bearers were blemished, ritually polluted and to be avoided, especially in public [2].

Individuals or groups who are actually observed or merely presumed to be experiencing the physical, behavioural, emotional or cognitive symptoms and signs of "mental health difficulties" (MHDs: e.g., addictions) of any type, complexity, intensity or duration [3] are often stigmatized on these bases by individuals, groups or institutions [1,2,4-6]. A quasi-systematic review of national, regional and local survey data, which were reported in 1990–2004, has confirmed that a substantial number of members of the public hold prejudicial views about those who experience MHDs [7].

Stigmatization manifests as discriminatory attitudes, stereotypes, labels and behaviour, which in devaluing, discrediting, marginalizing, disempowering or excluding and rejecting individuals, can produce harmful consequences (e.g., exacerbation of MHDs; unwillingness to seek help; withdrawal; feeling shame; self-blame or self-harm) [1,2,4-6,8-20]. While often afforded by a lack of valid knowledge about or exposure to individuals with MHDs [7], such bias can appear to be self-focused or may instead be thought to exemplify the dynamic of "anticipated discrimination," stem from associations with significant others who experience MHDs, or result from interactions with the helping professions [8,21].

Likely owing to differences in the characteristics of respondents as well as in study objectives and methods, which includes the contexts about which respondents were queried, no consistency has been observed in the estimated proportions of individual who have identified their MHDs as the reason for their having been discriminated against [9,21-23]. One rate reached 70% [9]. Yet, no proportion was obtained from a large scale, population study, and no investigation directly estimated the larger, societal consequences of MH stigma (e.g., lost productivity).

There have been calls worldwide to eliminate and prevent mental health stigma and its antecedents [11,12,18,24-28]. Canada's new Mental Health Commission considers this one of its highest immediate priority areas [29].

Various interventions have been developed and implemented to eliminate and prevent this discrimination [8,11-15,30]. We will argue later that the most effective and efficient strategies entail both "early" and ongoing, curriculum-based implementations of developmentally-appropriate, school-based interventions. At least in the developed world, schools afford continuing access to the largest gatherings of typically receptive, younger citizens. Moreover, even the youngest can stigmatize peers [8,10-13,31-33], although for other children such interventions would likely constitute primary prevention.

After confirming the absence from the published literature of a similar investigation, we conducted a systematic review of the scientific evidence concerning the benefits and harms of school-based interventions, which were directed at those 18 years of age or younger to eliminate or prevent MH discrimination.

## Methods

### Search Strategy

With input from a MH expert, the search strategy identified reports characterized by any language of publication or publication type [34]. Retrieving relevant, unpublished reports could help minimize the impact of a bias against publishing null or negative results. Various electronic databases were searched using a combination of subject terms, index terms and text words: Medline (1966 – January Week 1, 2007); OldMedline (1950 – 1965); PsycINFO (1806 – January Week 2, 2007); ERIC (1966 – December 31, 2006); Embase (1980 – January Week 2, 2007); CINAHL (1982 – December Week 2, 2006); the Cochrane Library, which included the Cochrane Central Register of Controlled Trials, DARE, and Database of Systematic Reviews (4^th ^Quarter, 2006); and, The British Education Index (December 31, 2006).

Additional data sources included reference lists of relevant reports that were searched manually, as well as key experts, organizations, and web sites (list available upon request). After duplicate citations were removed (Reference Manager11^®^), a final set of 6,341 unique citations had their bibliographic records (i.e., citation, key words, abstract) uploaded to systematic review management software (SRS, Version3^®^) and reviewed.

### Eligibility Criteria

Predefined eligibility criteria identified as relevant any school-based intervention (i.e., all types, materials, activities, clinical contents, complexity or duration), whose implementation to eliminate or prevent mental health stigma with students 18 years of age or younger was evaluated empirically using any research design, sampling strategy, number and timing of assessments, and stigma-relevant outcomes reflecting possible benefit or harm (e.g., attitudes, stereotypes or behaviour).

### Selection Process

Each application of eligibility criteria entailed a calibration exercise and a form that was developed and tested especially for this review. Two reviewers independently appraised each bibliographic record. Reports whose records passed this first screening were retrieved and evaluated independently by two reviewers. Reports were not masked given the equivocal evidence regarding the benefits of this practice [35]. A third screening was conducted by two independent reviewers to identify reports that presented data separately for our population of interest. Disagreements arising at screening levels 2 or 3 were resolved by forced consensus and, if necessary, third party intervention.

### Data Abstraction

Using a "single abstractor, single verifier" model, data were abstracted independently by five reviewers and placed directly within tables created specifically for our review. Disagreements were resolved by forced consensus and, if necessary, third party intervention. Data included results, reasons for losses to follow-up, and key characteristics of the intervention (e.g., objective; sequence and identity of the materials, activities and contents), population (e.g., sample size; age; cognitive-affective capacity to appreciate the intervention), and evaluation strategy (e.g., research design). When a study was described by more than one report, data were abstracted from all documents.

### Evidence Synthesis

**Figure 1:**
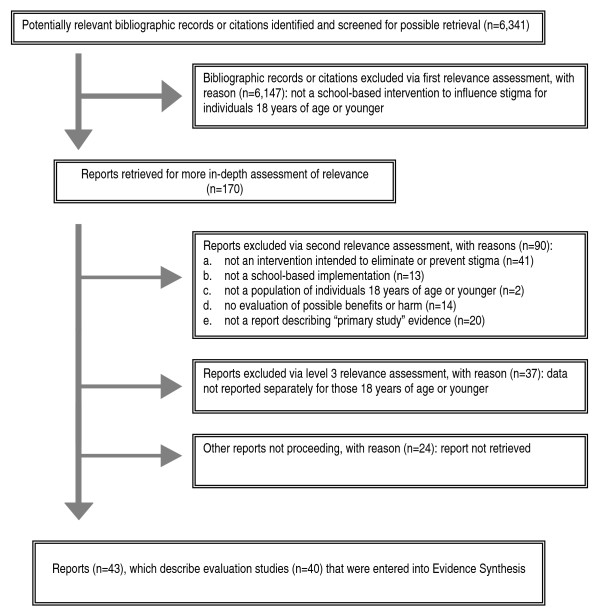
Enhanced QUOROM Flow of Evidence.

An enhanced QUOROM scheme represents the final status of each piece of evidence subjected to systematic review (Figure 1) [36]. Its description is followed by a qualitative, or descriptive, synthesis – with critical appraisal – of observed patterns of similarity within and covariation between study results, methods and populations. This facilitates the identification of "strengths" or "gaps" in knowledge about which factors are necessary or sufficient to reliably produce or preclude effects. Variables are also highlighted (e.g., pre-study exposure to stigmatization), whose possible, likely or known confounding influences were not adequately controlled for experimentally or analytically, and which require control in future efforts.

Interventions are organized by type, research design, and the appropriateness of controls (Additional file 1). The latter two factors together suggest the likelihood of being able to unequivocally attribute (no) effects to an intervention. Largely due to its inherent ability to control for selection bias and unknown sources of confounding [37,38] – and notwithstanding the need to assure its quality in other ways (e.g., appropriate controls; adequate control of known confounders) – the randomized controlled trial (RCT) is the research design best suited to establish an intervention's effectiveness [37]. Therefore, RCT evidence carried the greatest interpretative weight for us, and only these data were eligible for meta-analysis. Our decision to forego formally assessing the quality of individual studies is described below.

## Results

Of 6,341 records entered into initial relevance screening, 6,147 were excluded (Figure 1). All but 24 of the remaining 194 records had their reports successfully retrieved and subjected to a second, in-depth screening. This assessment excluded 90 reports, and a third disqualified 37 reports because data were not reported separately for our population of interest. Overall, 43 relevant reports were identified, which described 40 evaluation studies conducted worldwide (Additional file 1). Each of several reports described more than one study [11,12,39], and some studies were highlighted in more than one report (Additional file 1). Most reports were published in English-language journals; a few required translation into English [40-43].

We were unable to identify either the number of unique interventions within the evidence base or the exact number of unique implementations for some of them [11,12,44,45]. These observations demonstrate the "poor reporting quality" problem, which is described below.

Nevertheless, three intervention types were identified. The "education-only" type employed components (e.g., activities, events or materials such as a video), whose contents (e.g., stigmatizing attitudes toward MHDs, help-seeking or MHD care or professionals) were intended to be educational. The "contact-only" kind involved study participants having direct contact with someone(s) experiencing MHDs, who typically recounts their personal story about MHDs, help-seeking or receiving care, and may interact with students. The contact could also or instead be with self-identified representatives of the MH professions if the study focused on reducing the stigma related to help-seeking. An "education+contact" intervention included at least one component of each type. Almost all of the interventions included at least one education component. No intervention had as its central aim to foster protest about issues relating to MH, MHDs or related stigma [5].

Overall, investigators' choice of specific intervention types, interventions and their characteristics, implementation strategies, and study outcomes for use with study populations seemed realistic given stated study objectives. Most of the interventions appear to have been brought in from outside the school and its curriculum.

With rare exception, researchers conducted short-term evaluations of brief, single opportunity interventions (e.g., part of a day) conducted under naturalistic conditions (e.g., classrooms) [31,33,40,44-55]. Few interventions were implemented over several weeks or months [32,56-58]. Only Voeltz's multi-year, multi-semester contact-based programs might be considered somewhat intense [59,60].

The most frequently portrayed MHD in education-only interventions was depression; depression and schizophrenia were combined most frequently. Due to the preponderance of World Psychiatric Association (WPA) interventions, which represent their "Open the Doors" campaign, schizophrenia was most often highlighted within education+contact interventions.

Across intervention types, most participants were high school students. Yet, the age or grade levels of participants within educational interventions differed somewhat from those receiving education+contact ones. Participants as young as five years of age [31] or as early as grade one [31,32] were enrolled in educational efforts. No education+contact study appeared to enrol participants under the age of 12 years; some preadolescents might have participated in contact studies [59,60]. Three studies also reported data for participants who were slightly older than 18 years of age [43,57,61].

For each intervention type, evaluation strategies exhibited considerable variation in their scientific rigor. Both controlled and uncontrolled studies were conducted, which included a few RCTs as well as designs whose decreasing inherent ability to control for key sources of (e.g., selection) bias increasingly precludes the identification of reliable links between interventions and effects. Notable flaws characterized each design type and many other research methods (see below). Comparisons between interventions within controlled studies varied notably.

Outcomes differed in terms of their focus (e.g., attitudes, behaviour), content (e.g., MH, or MHDs or related stigma), and whether validated measures were employed. Educational and education+contact interventions focused primarily on knowledge, attitudes or stereotypes, with some concern for participants' behavioural intentions toward those experiencing MHDs (e.g., social distance). The possibility and nature of extra-intervention contact with those experiencing MHDs was not assessed for educational interventions. Some education+contact studies assessed self-reported, extra-intervention direct contact [39], attitudes toward MH professionals [51], and help-seeking attitudes or intentions [50,51,62]. No study explicitly aimed to influence the experience or effects of stigma arising from interactions with MH professionals. Possible consequences include erecting barriers to help-seeking [19].

Investigators rarely reported an intention to identify possible harms resulting from interventions. This observation holds for all intervention types and research designs. Where data were provided, there was no evidence to suggest that participants developed the type of serious negative self-scrutiny that can result from attempts to increase awareness (e.g., eating disorders) [63]. Instead, on rare occasions an intervention appeared to intensify stigmatizing attitudes (e.g., restrictions placed on others) or language (e.g., "dangerous"), or accentuate the differences between participants and those experiencing MHDs (e.g., autism) who were the "others" highlighted by the intervention [33,43,48,57]. However, an absence of descriptions of harm cannot be taken to indicate its absence.

For various reasons – which include the failure to conduct RCTs, to employ appropriate control groups (e.g., "no-intervention controls") and to adequately control (by design or analysis) both across and within study groups for confounding from pre-study or on-study influences – our systematic review did not identify one, even remotely ideal investigation whose results regarding possible benefits we can confidently accept as being reliable and valid. Extremely rare were studies that utilized methods we consider adequate (see below). This observation likewise applies to the few RCT investigations.

Only two [33,46] of five RCTs [33,39,46,58,64] employed appropriate "no-intervention control" groups. In having "control" subjects merely follow their regular school routine, three RCTs failed to control for various generic factors that define the receipt of any "active" intervention (i.e., a novel event; time extension; attention paid to participants; availability of information) [39,58,64]. Data generated by these control subjects cannot be meaningfully compared with data obtained from those who received the active intervention. Results from these studies are therefore at best only negligibly more revelatory than results achieved by uncontrolled investigations. That said, the two appropriately-controlled RCTs investigated different educational objectives, study populations, comparisons of interventions, foci on MHDs or outcomes; and, neither produced statistically significant effects, which affirm the benefits of their intervention (e.g., stereotypes; social distance: Additional file 1) [33,46].

Quasi-experimental designs lack RCTs' *inherent *potential to reveal unequivocal answers to questions of intervention effectiveness. That said, RCTs' problem concerning controls characterized eleven of 13 such designs [11,12,40,50,51,55,57,59,60,62,65]. Across intervention types only two employed appropriate "no-intervention controls" [66,67]. Two studies also employed appropriate "active" controls [59,60], and four *exclusively *enrolled "active" controls [11,43,48,49].

As with RCTs, quasi-experimental studies lacked comparability. They investigated different intervention types, objectives, populations, comparisons of interventions, illustrations of MHDs, and outcomes; moreover, the appropriately-controlled efforts revealed inconsistent evidence regarding benefit (e.g., attitudes) [11,43,48,49,66,67].

We do not highlight results from uncontrolled, pre/post or post-test only studies since their prominent, inherent vulnerability to threats to internal validity makes them unsuited to resolve our research question [44]. Furthermore, these studies exhibited a marked lack of comparability (e.g., objectives, interventions, populations, outcomes), in addition to inconsistent effects.

A paucity of comparable, soundly conducted RCTs prevented us considering conducting meta-analysis. What follows is a qualitative evidence synthesis, which highlights some of the sources of important between-study heterogeneity (i.e., interventions, controls, populations, outcomes). For two reasons a formal assessment of individual study quality (e.g., reporting clarity; internal validity) was not undertaken. Our qualitative synthesis includes a comprehensive critical appraisal of studies, and a time-consuming, formal assessment would be unlikely to meaningfully increase our appreciation of study limitations or how they differentiate studies.

The evidence displayed five important limitations, which constitute barriers preventing us drawing conclusive inferences regarding the benefits and harms of interventions and intervention types. The first barrier is the plethora of "gaps" in known characteristics of the interventions, populations, implementations, evaluations, outcomes and results. The many missing, unclear or contradictory data indicate poor reporting quality, which seriously hamper attempts to make sense of and reproduce these efforts.

Some reports failed to provide details regarding the intervention activities, materials or content [48,51]. With coordinators free to use materials from the "Open the Doors" program to suit their needs [11,12,43,67], and in the absence of better descriptions, we could not assume the equivalence of most WPA interventions. As well, almost no reports established interventions' developmental-validational history, or what was done to adapt them for use with their samples (e.g., pilot-testing) [11,31,32,43,45,65,68]. Occasionally, it was difficult to determine how interventions were implemented, implementers' identity or their required preparation. Many reports even failed to adequately describe those populations eligible for enrolment (e.g., age), study enrolees, study completers, and those lost to follow-up (with reasons) [32,39,40,48,49,52,56,59,60].

How evaluations and analyses were conducted was often difficult or impossible to discern. This included the identity and psychometric properties of measures, which confounders were controlled for, and whether an analysis was conducted for the intention-to-treat population or according to the research design (e.g., RCT) rather than for some subset of data (i.e., pre/post data for one study arm) [43,45]. Outcome data from multiple implementations across one or more sites were sometimes combined, yet described without explicit assurance that either the interventions or populations were comparable [11,44,45].

A dearth of adequate, RCT investigations is a second barrier. Yet, the paucity of these gold standard designs cannot be explained by ethical or scientific barriers inherent to our topic [33,39,46,58,64]. Likelier hurdles are a lack of methods expertise and funding. This barrier is significant since, in failing to control for selection bias, results from lesser designs can be swayed easily by factors such as motivation. Volunteers' enthusiasm, for example, can affect study performance [39].

Possibilities of such bias were acknowledged by some investigators who had conducted quasi-experimental studies. One study allowed participants to select their study projects [67]. In another, teachers who did not volunteer to participate in an intervention, but who were then asked to receive a control intervention, may have had their lower enthusiasm for participation in the study directly affect outcomes [51]. Investigators conducting an uncontrolled pre/post study also noted that teachers' self-selection as implementers of their intervention may have influenced outcomes [44].

Flawed research methods, which characterized all research designs, define a third barrier. Notwithstanding poor reporting practices, often enough there were sufficient details to indicate many problems. These include: interventions that were recognized by investigators as having been too brief and lacking in continuity of exposure to make even a short-term difference [33,39,46,64,66]; interventions lacking the concreteness, salience or realism apposite to participants' specific developmental levels [33,50]; inappropriate "no-intervention controls;" unvalidated outcomes, and failing to pre-establish validated "meaningful clinical changes;" short-term assessments; and, neglecting to analyze data from the intention-to-treat population as well as failing to interpret results in light of data concerning reasons for losses to follow-up.

Perhaps the most profound lapse is most studies failed to explicitly consider that what participants bring to an intervention can significantly affect outcomes. One's readiness to appreciate and benefit from an intervention is shaped by past experience and needs to be taken into account. Otherwise, this factor can seriously compromise attempts to attribute (no) effects to the intervention.

Foremost among these characteristics is participants' prior exposure to individuals experiencing MHDs or related stigma. Those with and without previous experience may respond very differently to a stigma-focused intervention due to differences in heightened empathy. But, virtually no studies assessed, then controlled for this factor in their research design or analytically. Moreover, the failure to explore data separately for those participants in a study who did and did not exhibit elevated pre-study stigmatizing perspectives or behaviour means that the effectiveness of an intervention as secondary and primary prevention, respectively, could not be ascertained.

The marked inconsistency in the approach to investigating the effects of interventions is a fourth barrier. The lack of comparability is observed for each study parameter, which include the definition of the objectives, interventions (e.g., activities, materials, content), controls, populations, implementations, evaluations, outcomes, (validated) instruments, analyses, and control for confounders (Additional file 1). Without methodologically-sound replication attempts, the evidence cannot unequivocally attest to or reject the value of any single intervention. The absence of effects observed within the rare, appropriately-controlled RCTs [33,46] and the inconsistent results revealed by the appropriately-controlled quasi-experimental designs [11,43,48,49,66,67] together constitute a fifth barrier preventing us drawing conclusive inferences.

## Discussion

Our systematic review identified scientific evidence concerning the benefits and harms of school-based interventions, which were directed at students 18 years of age or younger to influence MH discrimination. However, five limitations prevent us drawing conclusive inferences regarding interventions' risk-minimizing ability to eliminate or prevent it.

Consequently, we cannot determine which interventions or intervention types "work," which "work best" or "better," for whom, in what terms (i.e., outcomes) or under what conditions (e.g., setting). Likewise, we cannot identify the intervention types, activities (e.g., contact), materials (e.g., video) or contents (e.g., MHDs portrayed) that are necessary or sufficient to produce population-specific or population-independent benefits while also avoiding harm. Few studies reported having investigated possible harms. As a result, we cannot recommend any single school-based intervention or intervention type.

Yet, the evidence also does not permit us to identify those intervention types, interventions or characteristics that lack the potential to reliably produce benefits. Virtually none of the efforts to date have entailed appropriate appraisals of this potential. New research should likely begin by correcting this state of affairs. There is likely little sense in trying to "reinvent the wheel" when there are many approaches, whose value is largely unknown. Moreover, there are numerous school-based interventions, whose impacts have not yet been evaluated [11,69-77]. Yet, going beyond existing philosophies and practices should not be ruled out.

Additional recommendations for future research are informed by largely consistent viewpoints obtained from three sources (see below): a) investigators' interpretations of their and others' suggestive yet inconclusive results from studies that we reviewed; b) researchers whose efforts to influence MH stigma focused on individuals outside our population of interest; and c) those youth, service users, advocates, volunteers, researchers, educators, clinicians, and policy-makers who attended a recent international workshop [78]. The workshop was organized in response to the findings of our review, and was intended to derive a meaningful research agenda by further examining the state of the science from the perspective of "real world" experience and expertise. Foremost among the shared views is that "contact" is likely necessary but alone may not be sufficient to produce maximally beneficial outcomes (see below).

What we propose cannot be drawn solely from those interventions, which our review highlighted, since virtually none entailed ongoing (e.g., multiple exposures), curriculum-based efforts. Rather, they tended to be one-off, brief events whose typically cognition-focused outcomes (e.g., knowledge, attitudes, stereotypes) were evaluated over the short-term in mostly older children; and, many did not aim to facilitate an experiential engagement with individuals experiencing MHDs.

It is our view that interventions should be employed to develop a sustainable, self-regulating "compass," which by its very nature makes highly unlikely, if not impossible, any inclination to perceive or behave in ways that discriminate against those experiencing MHDs. But, to achieve this aim, school-based interventions should likely exhibit certain characteristics. Otherwise, we expect that any changes brought about by strategies that do not satisfy these conditions will not be substantive enough to assure their generalization much beyond the specific context or time period in which they were implemented, or the developmental stage of those students who were exposed to them.

Interventions should likely involve experiential activities, which in facilitating students' interaction with other human beings, engage students' feelings and behaviour, not just cognition-based points of view [33,45,50,79,80]. As well, given how early in life discriminatory viewpoints and behaviours can appear, early intervention is a reasonable aim [10,32,66]. While for some students this might constitute primary prevention, for others it would represent secondary prevention.

The intervention should likely be implemented multiple times within and across the school years (e.g., "critical periods") [40,43,50,51,59,65]; and, starting early could maximize the number of exposures to activities, materials and contents that are repeated both within and across successive stages of cognitive and affective development. Yet, to be able to foster a deepening integration of the benefits afforded by these exposures, the activities, materials and contents should be modified in ways, which over successive implementations, are incrementally challenging yet always developmentally-appropriate. In short, we propose a curriculum, whose implementations reinforce and build upon prior ones [32,45,67].

Implementers, who include those experiencing MHDs, should likely be those with whom the children or youth are most likely to identify (e.g., those most similar to themselves) [50,67]. Yet, actively involving their teachers, other school staff, the school administration and parents could maximize the likelihood of making a sustainable difference. Moreover, child and adolescent involvement in creating, refining and pilot-testing the curriculum is likely essential to maximize the relevance and developmental appropriateness of its components and the timing of their implementation.

Our review team echoes what other sources have opined about the need for contact-based interventions, which reflect an experiential approach, to produce substantive, especially behavioural, change [39,40,43,51,52,62,65,68,78,79,81-83]. For example, a recent, large and well-controlled meta-analysis of study data, which were identified without specific reference to our topic, found that intergroup contact typically reduces intergroup prejudice across a broad range of groups and contexts [84]. Yet, while the creation of carefully structured contact situations enhanced the magnitude of benefits, it was not required to produce them.

As well, some contact-based interventions, whose evaluations we appraised, did produce some suggestive evidence of benefit, which might be confirmed by high quality RCTs [43,48,49,67]. Perhaps most notable, however, are the conclusions offered by investigators, whose education-only interventions we reviewed. Several claimed that contact is likely necessary to produce substantive change [40,65]. Unsurprisingly, contact's candidacy as the most promising way to do so also comes from the observation that research on a frequently employed alternative has not engendered confidence in the latter's capacity to reliably produce such benefits. Education-only strategies sometimes produce shifts in knowledge, attitudes or stereotypes [85], yet these do not reliably predict behaviour (see below) [33,40,46-48,50,60,67].

It is our view that the greater promise of contact interventions to produce substantive change stems from the assumption that, compared with approaches that typically aim to influence responses to short-term, post-intervention queries concerning attitudes, stereotypes or knowledge, they are more likely to kindle the development of empathy. Our review did not identify a single contact strategy that was explicitly evaluated for its impact on empathy as an intermediate outcome, yet we hypothesize that empathy is the mechanism by which contact can produce substantive, behavioural change [33,50,86-89]. We also posit that the development of empathy, which might underlie a self-regulating "compass," likely requires the stimulation of affect and affect-based understanding within contact scenarios.

A recent controlled study conducted outside a school context, which involved naturalistic contact, found that stimulating affective responses can yield prominent change [79]. Pairing volunteers with individuals, who were experiencing severe MHDs, reduced negative affective responses in the former with reference to the latter; and, changes in affective response were directly related to the quality of the contact. Furthermore, Angermeyer and Matschinger found that the tendency for pro-social action toward those individuals experiencing MHDs depended on emotional reactions [80]; and, personal stories shared by those experiencing MHDs can produce an affective response in children and youth [33,50].

Authors of a study we evaluated observed that, when asked to select less stigmatizing descriptions, study participants tended to focus on others' feelings [45]. Evolutionary theory notes that the perception of the emotional state of another living being appears to automatically activate the creation of a matching emotional state in the observer [90]. Then, with increasing cognitive capacity, "state-matching" – which underlies the fundamental, nonverbal experience of empathic identification – appears to evolve into more complex forms, which include concern for the other and perspective-taking. Whether, and how, "mirror neurons" play a mediating role in state-matching for different species remains to be confirmed [91]. Nonetheless, at least for human beings, the nature of the changes brought about by contact can also involve a notable perceptual-cognitive shift in how we typically experience reality and self.

The distinctions and dualities produced by our natural capacity to "difference-make" are the hallmark of the lifelong project by which our sense of personal (or collective) identity (i.e., self-sameness) develops and is (pre)served, and they allow us to make sense of reality and self [92-98]. However, especially unself-reflective use of this capability can culminate in the typically fear-imbued identification of "others" from whom separation by way of acts of exclusion (i.e., boundary-making) comes naturally, and against whom self-(pre)serving, discriminatory attention can be directed with little conscious effort [92-94,98].

That said, our equally natural capacity for empathy affords an experience of experiential identification and understanding ("resonance"), which allows the observer to transcend the presumed boundary between self and "other" and thereby makes it more difficult to (exclusively) relate to this "other" in ways characterized by exclusion or exclusivity [98]. Empathy may therefore be thought of as promoting an experience of inclusion and inclusiveness, which typically entails noticing, and acting upon the recognition of similarity or sameness. This capacity can balance our inclination to notice, and act based upon, difference at the same time that it can cultivate a sense of community. Finally, the development of empathy is likely integral to the realization of one facet of our transpersonal human potential, which is the compassion that knows neither boundaries nor "others" [95].

So, how might an empathy-facilitating, contact-centered curriculum be described? Each implementation could involve students being brought into direct contact with those experiencing MHDs. However, based on work which has not had our population of interest as its explicit or sole focus, it has been suggested that certain criteria should be met in order for contact to stimulate meaningful change [81,82].

Interventions should promote interactions between those experiencing MHDs and their "audience," which demonstrate their equal status, provide an opportunity for them to get to know each other, and foster their active co-operation in the pursuit of a mutual goal such as sharing information that challenges negative stereotypes [81]. Yet, a narrative review of that literature found that many of the studies from which these criteria were derived were fraught with the same methods-related problems that we observed in our review [83]. As an aside, failing to satisfy some of these criteria may explain why the behaviour of some MH professionals (e.g., "playing the elevated, expert interpreter and labeler of others' experiences and reality") [99,100] can be seen as stigmatizing by those who experience MHDs [82].

The typical contact approach, which invites into the classroom those who reflect upon their experiences with MHDs, is likely too large a cognitive challenge for the youngest students (e.g., those in kindergarten or the first few grades). Instead, a generic form of contact could be employed, which aims to stimulate and develop empathy. Only as these children develop would the typical types of contact intervention be employed.

An exceptional candidate for inclusion in an empathy-centered curriculum, which has been successfully implemented as early as kindergarten and as late as early high school, is the Roots of Empathy program [86,89]. It does not assert as one of its objectives the elimination or prevention of MH discrimination. Rather, it aims to cultivate the development of empathy and emotional literacy, to reduce levels of bullying, aggression and violence, and to promote pro-social behaviour, among various civility-related aims.

The program brings a neighborhood infant and parent into a classroom every three weeks for one school year. Using a structured, manual-based curriculum, which describes themes that are broken down into four age ranges, a trained instructor coaches students to observe the baby's development and label the child's feelings. Controlled, prospective studies have shown a significant decrease in aggression and bullying, along with an increase in pro-social behaviour [86,89]. The program appears to promote certain "positive" facets of MH. These benefits have been observed immediately following completion of the program, and some were maintained after three years.

But, its usefulness in developing social and emotional learning could contribute either directly or indirectly, through its impact on intermediate conditions such as social inclusion and inclusiveness, to the prevention or elimination of various forms of (e.g., MH) discrimination. Pilot-testing should reveal how, when, and if this program could become a part of a curriculum that strives to achieve these aims. But, even if it were found to be an essential component, any plan to directly address MH discrimination would likely need, at some point in the curriculum, to employ contact elements that incrementally and appropriately expose students to individuals and issues with a specific focus on MH, MHDs and related discrimination. Contact opportunities might benefit from establishing certain favorable conditions (e.g., equal status).

Activities, materials and contents could be modified in ways that deepen and extend the development of empathy. For example, exercises (e.g., perspective-taking) could make increasingly explicit the relevance of empathy for dealing with issues of MH, MHDs and MH discrimination. Modeling and role-playing [33,39,51,60] might be used to explore and practice appropriate ways to express empathy-guided behaviour.

Developmentally-appropriate discussions could be scheduled strategically over the years, which successively focus attention on issues concerning: a) difference-making, which involves the identification of differences (e.g., "bad" *versus *"good") integral to acts of (social) exclusion and discrimination; b) the perception of sameness, which can be associated with acts of (social) inclusion; c) MH and wellness (e.g., resilience); d) MHDs and help-seeking; and, e) starting with older children, the power dynamics of MH discrimination. Engaging their conceptual frameworks would depend upon children's cognitive and affective readiness. Educational materials, whose nature would need to be determined, could also be used to reinforce and extend issues raised through contact (e.g., focusing on similarities) [67]. Both "heart" and "mind" could be educated concurrently within the curriculum [101].

Any resistance by teachers or schools to the inclusion of such a curriculum could be resolved by recognition of the following. Since what it aims to achieve with reference to MH discrimination employs at least one element (e.g., developing meaningful consideration for others) that is represented somehow within some, extant school (e.g., "anti-bullying") programs, it might be possible to unify these efforts. A single curriculum could be developed, which fosters the development of empathy, and which in promoting social inclusion and inclusiveness, serves to prevent or eliminate various forms of negative attention that can be directed at "others" (e.g., racism, ageism, sexism, MH discrimination, despoiling the environment). Lastly, getting different sectors (e.g., MH, health, education, youth justice) to recognize the potential of an empathy-based curriculum, which concurrently or sequentially influences unique and overlapping outcomes of pertinence to their respective mandates, might be enough to get these stakeholders to meet and collectively support its use.

This discussion, when seen against the backdrop of our review, suggests several questions that could be investigated in future research. Given our hypothesis that school-based interventions require empathy-inducing contact in order to produce substantive change, and the recognition that education-centered components at least in principle could contribute to eliminating or preventing MH discrimination, we might ask whether education meaningfully enhances any of the benefits that might be produced by contact alone. It may be the case that contact is necessary to produce substantive change yet alone it may be insufficient to do so. To bring about this change, education may be needed as an add-on. Yet, this view suggests that we already know that contact alone reliably produces meaningful benefits. We do not know this.

Therefore, several questions require investigation: Does contact (C) produce substantive changes, whose nature and magnitude are pre-established? Does education (E), when added to contact (C), produce substantive changes, whose nature and magnitude are pre-established? Does adding education to contact (C+E) produce significantly greater change than that produced by contact (C) alone? These questions may be investigated concurrently within a single research design. But, before we describe it, we turn our attention to the issue of appropriate outcomes.

Changes in knowledge, attitudes and stereotypes do not reliably predict behaviour [33,40,46-48,50,60,67]; and, when evaluated especially in the short-term, these outcomes appear to be vulnerable to socially desirable response sets shaped by expectations that can be readily communicated via the nature of the intervention or the contents of pre-intervention assessments [33,39,40,60,64]. Moreover, it is discriminatory action or inaction that is particularly noxious. Consequently, the primary outcome should be behaviour, which can be observed especially under naturalistic conditions. Virtually no studies that we identified utilized such outcomes [39].

The outcomes also need to reflect the kinds of activities, materials and contents that hitherto have been provided. For younger children, schoolyard play, which focuses on the inclusion/exclusion of peers who are perceived to be different on some or any basis, could be assessed. Starting with older children, who are exposed to individuals and issues that focus on MH, MHDs or related discrimination, acts of inclusion/exclusion could be assessed with reference to others whose differences are defined by their MHDs.

Secondary outcomes might elucidate the perception of social distance, which as a possible measure of inclusiveness, could capture students' expressed inclinations to interact with those who are different in some way (i.e., for the youngest children) or specifically because they experience MHDs (i.e., for the oldest children). One subtle way to evaluate respondents' intended behaviour might be to ask them to describe their peers' intended behaviour toward someone experiencing MHDs. These observations, which in one study pertained to a child with autism, better predicted students' behaviour than did descriptions of their own intended behaviour [33]. The latter may be more susceptible to socially desirable responding. Finally, the judicious inclusion of a few tertiary measures such as knowledge, attitudes and stereotypes could allow us to begin to identify the possible causes or correlates of (failed) behaviour change.

Whatever the outcomes, they should be validated as well as developmentally-appropriate. At what time intervals or developmental levels they should be assessed within the curriculum would need to be determined via pilot work. Finally, possible harms require a direct yet potentially subtle and qualitative evaluation [51,66,102] since efforts to prevent harmful events can inadvertently produce them [33,63]. Harms data were under-evaluated within our evidence base, although unidentified harms could account for some studies' notable numbers of withdrawals or dropouts [40,57,65].

Returning to the proposed research, pilot work could justify conducting a two-group RCT (i.e., C+E *versus *C). Independent, uncontrolled before-after designs would have to demonstrate that each intervention can produce predefined types and magnitudes of benefit as well as minimize harm. As stated earlier, our identification of five RCTs suggests the absence of ethical or practical barriers to conducting these designs to investigate our topic [33,39,44,46,50,57,58,64,66].

The most appropriate design is the cluster RCT, whereby schools that exhibit similar profiles of relevance to the study (e.g., same basic curriculum; presence of children with MHDs) would be randomized to study arms to minimize the contamination that can arise when students within the same school are allocated to different study groups and inevitably discuss their respective exposures [64]. Contamination can wash out real effects [40,58,59].

Yet, other criteria require satisfaction in order to allow us to meaningfully attribute any (lack of) observed benefits exclusively to an intervention. While educational activities, materials or contents would be used to complement contact (i.e., C+E), those participants in the condition (i.e., C) that does not receive them would need to have something provided in order to control for the "generic" elements (e.g., attention paid, time spent, a novel event) that are given to the C+E group. Attention could be directed to general health issues.

Outcomes would be assessed at baseline as well as following interventions in order to determine whether, in addition to between-group differences, meaningful changes occur within each study group. One possible result is that both interventions produce the same magnitude of meaningful benefit, which would allow schools to select between two types of curriculum (i.e., C+E *or *C).

Since pre-intervention exposure to those with MHDs, a personal history of MHDs and help-seeking, prior experiences of MH discrimination, pre-study levels of empathy, baseline cognitive-affective capacity, and pre-intervention types and intensities of discriminatory knowledge, attitudes or stereotypes about MH can each influence how students respond to a review-relevant intervention, these variables require pre-study evaluation (and possible experimental control) [11,33,39,40,43,44,46,48,50,53,58-60,62,64,66]. The presence and absence of notable pre-study biases would identify those students for whom the intervention is secondary and primary prevention, respectively [33,39,44,46,60,67].

However, since evaluating pre-study characteristics can serve as cues that sway responding in expected or desired directions as well as establish a ceiling on the magnitude of possible changes from baseline, it might be best to conduct pre-study assessments for only a randomly selected half of the schools that are allocated to each study condition [50,67]. Participants at the other schools would be asked about contents outside the study's focus (e.g., general health). This strategy would permit an evaluation of the impact of conducting pre-study assessments on results.

Primary analyses would be completed with data obtained from the intention-to-treat population, while the impact of potential, confounding factors could be investigated within secondary analyses. Additional, candidate confounders, which demonstrated some potential to influence outcomes within our evidence base, include: socio-demographic factors; religious beliefs; and, the presence at school of students who are experiencing MHDs [11,44-47,49,50,59,60,66,71-73].

Finally, it could be argued that, to be most salient for older children and youth, the MHDs portrayed via contact and education exposures should be ones that are the most prevalent within this population. Instead of focusing on schizophrenia, which is the case within the WPA program, interventions could highlight MHDs that students are more likely to encounter amongst peers, for example (e.g., anxiety disorders).

Limitations of our review include being unable to contact all authors to clarify poorly reported study details, although it is unlikely that successfully obtaining these particulars would have changed our observation that almost no studies exhibited even adequate methods-related quality. Our focus on students 18 years of age or younger means that we did not review studies that exclusively enrolled older students. A list of these studies is available upon request.

## Conclusion

The identification of five limitations within the scientific evidence base prevents us drawing conclusive inferences concerning the value of school-based interventions to prevent or eliminate MH discrimination. Nevertheless, there exists enough suggestive evidence to inform a future research direction, which takes behavioural change as its primary outcome. Likely the most promising course involves developing a curriculum, which in being implemented early as well as repeatedly both within and over the school years, would employ a generic form of direct contact for the youngest children, followed by direct contact with individuals experiencing MHDs for older children. This should encourage the development of empathy and, in turn, an orientation toward social inclusion and inclusiveness. In this way, discrimination directed at others on the basis of their mental health might be prevented from emerging in some students' lives while for others, who already demonstrate such proclivities, it could be eliminated. To maximize the likelihood of identifying the value of such an approach, gold standard research designs and methods are required.

## Competing interests

The authors declare that they have no competing interests.

## Authors' contributions

HMS conceived and refined the research question, developed, implemented and supervised the implementation of the systematic review methodology, searched for and obtained relevant evidence, conducted relevance assessments, organized and conducted the qualitative synthesis and critical appraisal, and took the lead in preparing the manuscript. AG searched for and obtained relevant evidence, conducted relevance assessments, abstracted data and contributed to the qualitative synthesis. ML, DL, ABL and RG abstracted data as well as contributed to the qualitative synthesis. JVB developed, validated, conducted, organized and maintained the results of all formal searches within electronic databases. All authors interpreted the results as well as prepared the manuscript and its table and figure. All authors read and approved the final manuscript.

## Supplementary Material

Additional file 1 Table 1. Key Study Characteristics. The data provided represent the key characteristics of studies deemed relevant for the systematic review. Table 1 also includes reference to six companion reports of relevant studies [103-108]. Click here for file
