# Supplementary material for: Effects of school-based interventions on mental health stigmatization: a systematic review
Source: Child Adolesc Psychiatry Ment Health. 2008 Jul 21;2:18. doi: 10.1186/1753-2000-2-18 (PMC2515285; doi:10.1186/1753-2000-2-18)
Supplement: Additional file 1 — Table 1. Key Study Characteristics. The data provided represent the key characteristics of studies deemed relevant for the systematic review. Table 1 also includes reference to six companion reports of relevant studies [103-108]. [file 1753-2000-2-18-S1.doc]

##### Table 1. Key Study Characteristics

| **Author, Year, Location: Intervention** | **Research Design:**  **Comparison Groups**  **(n-enrolled/n-completed)** | **Age of Participants** | **Intervention Components** | **MH Topics Portrayed** | **Length** | **Outcomes** |
| --- | --- | --- | --- | --- | --- | --- |
|  | | | | | | |
| Education-Only Interventions | | | | | | |
| Chung, 2004, Hong Kong: Less Pejorative Chinese Label for Schizophrenia [46] | RCT (314/313): **E** (NR/NR) vs **E-ctrl** (NR/NR) vs **E-ctrl** (NR/NR) vs **No-E-ctrl** (NR/NR) | 15.2 (13-18) y (NBD by group) | I | Schizophrenia | NR | Social distance; stereotypes of schizophrenia; attributions of MI |
| Swaim, 2004, U.S.: Autism Video [33] | RCT (233/233): **E+E** (78/78) vs **E** (77/77) vs **No-E-ctrl** (78/78) | Gr 3: 9.1 (NR) y; gr 6: 12.0 (NR) y | V | Autism | NR (less than 20 min) | Stereotypes; willingness to interact; perceptions of similarity |
| Rahman, 1998, Pakistan: Rural MH Program [64] | Cluster RCT (100/100): **E** (50/50) vs **No-E-ctrl*** (50/50) | NR (12-16) y | I, L, W, A, R | NR | NR (4 mo) | Knowledge & attitudes about common MH disorders |
| Schell, 1999, U.S.: Educational Intervention [66] | Quasi-experimental (146/NR): **E** (NR/73) vs **No-E-ctrl** (NR/68) | NR (11-13) y | S, I, D, GE | Schizophrenia, depression, bipolar disorder | 3 55-min lessons (3 days) | Attitudes about MI |
| O’Kearney, 2006, Australia: *MoodGYM* [55] | Quasi-experimental (78/CT): **E** (40/CT) vs **No-E-ctrl*** (38/CT) | NR (15-16) y | I, V, Q, IE | Depression, anxiety | 5 30-60 min  (5 modules) | Depression; attributional style; self-esteem; attitudes about depression |
| Hebert, 2000, Canada: *Les prejuges… j’connais pas* [40] | Quasi-experimental (438/373): **E** (NR/214) vs **No-E-ctrl*** (NR/159) | NR (gr 11) | I, S, D, GE | Depression, manic depression, schizophrenia, OCD | 75 min (1 day) | Attitudes & opinions about MI; social distance; knowledge about MI |
| Petchers, 1988, U.S.: *After the Tears* [65] | Quasi-experimental (102/46): **E** (NR/NR) vs **No-E-ctrl*** (NR/NR) | 17 (NR) y | V, D | NR | NR  (6 lessons) | Knowledge & opinions about MH & MI |
| Essler, 2006, U.K.: Theatre Group [54] | Pre-post (104/CT) | NR (13-14) y | R, GE | MH problems | NR (2 phases) | Knowledge & stereotypes about MH problems |
| Stuart, 2006, Canada: *Reaching Out* [61] | Pre-post (CT/CT) | NR (≤13 - ≥18) y | L, V, D, R | Schizophrenia | NR (2 lessons) | Knowledge about schizophrenia; behavioral intentions; social distance |
| Okasha, 2005, Egypt: *Open the Doors* [11,12] | Pre-post (3000+/NR) | NR (high school) | NR | NR (likely schizophrenia) | NR | Knowledge about schizophrenia & treatment; attitudes |
| Lauria-Horner, 2004, Canada: MH Curriculum [32] | Pre-post (158/NR): Grade 1-3 (66/NR); Grade 4-7 (92/84) | NR (gr 1-7) | L, GE | Emotional development, depression, anxiety disorders, ADHD | 4 hr/wk (16 wks) | Knowledge & attitudes about MH |
| Watson, 2004, U.S.: *Science of Mental Illness* [44] | Pre-post (NR/1566) | NR (gr 6-8) | I, V | Depression, ADHD, schizophrenia | 4.5 hr (8 sessions) | Attitudes about MI & treatment |
| Pinfold, 2003, U.K.: *Open the Doors* Phase I [45,103-105] | Pre-post (634/472) NBD by phase | NR (14-15) y | V, L, GE, I, D | NR | 2 1-hr sessions (1 day) | Knowledge of MH facts; social distance; attitudes |
| Gudmundsdottir, 2001, Iceland: *Icelandic Mental Health Promotion Project* [53] | Pre-post (81/NR) | All pts 14 y | L | NR | NR | Knowledge of MH facts; attitudes about MH |
| Lake, 1989, U.K.: Mental Health & Stress [56] | Pre-post (18/13) | NR (6^th^ Form) | L, D, I | History of MH, human development, relationships, depression, psychoses, bereavement, stress, substance abuse, MH service providers | 7.5 hr (NR) | Knowledge |
| Morrison, 1979, U.S.: Demythologizing Seminar [47] | Pre-post (24/24) | 16.58 (NR) y | L, I | NR | 50 min (1 day) | Attitudes toward MI; fear of mental patients |
| Shah, 2004, England: Mental Illness Talk [31] | Post-test (NR/NR) | NR (5-11) y (split into 3 age grps) | S, D, GE, A, R (differed by age) | Bipolar disorder, depression, anorexia, signs of psychosis (differed by age) | 20-30 min (1 day) (differed by age) | Observations of reactions & changes in pts |
| Contact-Only Interventions | | | | | | |
| Slininger, 2000, U.S.: Physical Education Intervention [58] | RCT (NR/131): **C** (NR/49) vs **C** (NR/45) vs **No-C-ctrl*** (NR/37) | **C**: 9.35‡ (9-10) y; **C**: 9.35‡ (9-10) y; **No-C-ctr***: 9.5‡ (9-10) y | DC, GE, W | Children w/ severe MR | 25 min/day (4 wks) | Attitudes toward peers w/ disabilities; behavioural intentions |
| Voeltz, 1982, U.S.: *Special Friends* [59] | Quasi-experimental (NR/817): **C** (NR/241) vs **C** (NR/288) vs **No-C-ctrl*** (NR/288) | NR (gr 4-6) | DC, GE | Multiple handicaps, MR | 3+ semesters | Attitudes toward handicapped peers |
| Voeltz, 1980, U.S.: *Special Friends* [60] | Quasi-experimental (NR/1310): **C** (NR/433) vs **C** (NR/454) vs **No-C-ctrl*** (NR/423) | NR (gr 2-6) | DC, GE | Multiple handicaps, MR | 2 semesters | Attitudes toward handicapped peers |
| Education vs Contact Interventions | | | | | | |
| Sato, 2005, Japan: *Open the Doors* [11,12] | Quasi-experimental (NR/303): **C** (NR/NR) vs **E** (NR/NR) | NR (high school) | **C**: DC; **E**: L | Schizophrenia | NR | Social distance; knowledge |
| **Education+Contact Interventions** | | | | | | |
| McConkey, 1983, Ireland: Changing Perceptions [39] | Cluster crossover RCT (410/NR): **E+C** (197/NR) vs **No-E+C-ctrl*** (213/NR) | NR (15-17+) y | R, V, D, W, DC, GE, I, P | Mental handicap in adults | 6 40-min sessions (6 wks) | Type & frequency of contact w/ mentally handicapped people; use of terms; knowledge of causes; attitudes |
| Schulze, 2003, Germany: *Crazy? So What!* [67] | Quasi-experimental (150/150): **E+C** (90/90) vs **No-E+C-ctrl** (60/60) | **E+C**: 14.8 (14-18) y; **No-E+C-ctr**: 15.4 (14-18) y | DC, D, A, W, P | MI in general, schizophrenia | NR (5 days) | Stereotypes |
| Tolomiczenko, 2001, Canada: *Beyond the Cuckoo’s Nest* Adaptation [48] | Quasi-experimental (NR/575): **E+C** (NR/186) vs **E+C** (NR/214) vs **E+C** (NR/175) | NR (high school) | V, DC, D, L, GE | NR | 2 hr (1 day) | Attitudes about MI |
| Husek, 1965, U.S.: Mental Health Talk [49] | Quasi-experimental (498/NR): **E+C** (NR/NR) vs **E+C** (NR/NR) vs **E** (NR/NR) | NR (high school) | L, DC | MI in general | 20 min (1 day) | Attitudes about MI |
| Meise, 2000, Austria: *Schizophrenia Has Many Faces (Open the Doors)* [41-43] | Quasi-experimental (NR/NR): **E+C** (NR/57) vs **E** (NR/57) | 17 (16-19) y | V, D, L, DC | Schizophrenia | 2 hr (1 day) | Emotional reactions; attributions; social distance; attitudes |
| Buizza, 2005, Italy: *Open the Doors* [11,12] | Quasi-experimental (NR/NR): **E+C** (NR/186) vs **No-E+C-ctrl*** (NR/NR) | NR (high school) | L, V, I, DC | Schizophrenia | 2.5 hr (1 day) | Knowledge & attitudes about MI |
| Baumann, 2005, Germany: *(Open the Doors)* [11,12] | Quasi-experimental (NR/NR): **E+C** (NR/NR) vs **No-E+C-ctrl*** (NR/NR) | NR (14-18) y | DC, A | Schizophrenia | NR | Social distance |
| Rickwood, 2004, Australia: MI Education Program [50] | Quasi-experimental (NR/457): **EC** (NR/309) vs **No-E+C-ctrl*** (NR/ 148) | 16 (14-18) y | L, D, R, I, DC | Depression, schizophrenia, MI in general | 50-90 min (1 day) | Attitudes; social distance; knowledge; help-seeking intentions |
| Ng, 2002, Hong Kong: Training Program [57] | Quasi-experimental (219/169): **E+C** (117/79) vs **No-E+C-ctrl*** (102/90) | **E+C**: 15.04 (12-21) y; **No-E+C-ctr***: 14.96 (12-21) y | GE, L, D, DC, P | NR | 10 1-hr sessions (10 wks) | Attitudes about MI |
| Esters, 1995, U.S.: Psychoeducational Intervention [62,106,107] | Quasi-experimental (40/40): **E+C** (20/20) vs **No-E+C-ctrl*** (20/20) | **E+C**: 14.55 (13-17) y; **No-E+C-ctr***: 14.85 (14-17) y | V, D, I, L, DC | Affective disorders, schizophrenia | 3 90-min sessions (3 days) | Conceptions of MI; attitudes about psychological help-seeking |
| Battaglia, 1990, U.S.: MI Awareness Week [51] | Quasi-experimental (1662)†: **E+C** (1380)† vs **No-E+C-ctrl*** (282)† | **E+C**: NR (M gr = 9.9); **No-E+C-ctr***: NR (M gr = 9.9) | L, I, DC | Psychiatry, drugs, alcohol, suicide, depression | 45 min (1 day) | Attitudes toward psychiatrists & help-seeking |
| Arboleda-Florez, 2005, Canada: *Open the Doors* [11,12] | Pre-post (1534/607) | NR (gr 9 & 11) | L, DC | Schizophrenia | 60-90 min (1 day) | Knowledge about schizophrenia; social distance |
| Arboleda-Florez, 2005, Canada: *Open the Doors* [11,12] | Pre-post (318/251) | NR (gr 9 & 11) | L, DC | Schizophrenia | 60-90 min (1 day) | Knowledge about schizophrenia; social distance |
| Warner, 2005, U.S.: *Open the Doors* [11,12] | Pre-post (NR/NR) | NR (high school) | L, DC | NR | NR | Knowledge; attitudes |
| Pinfold, 2003, U.K.: *Open the Doors* Phase II [45,103-105] | Pre-post (634/472) NBD by phase | NR (14-15) y | V, L, GE, I, DC, D | NR | 2 1-hr sessions (1 day) | Knowledge of MH facts; social distance; attitudes |
| CAMH, 2001, Canada: *Beyond the Cuckoo’s Nest* Adaptation [76,108] | Pre-post (NR/278) | NR (secondary school) | NR | NR | NR | Knowledge about MI; attitudes |
| McConkey, 1983, Ireland: Changing Perceptions [39] | Pre-post (368/NR) | NR (5^th^ Year) | R, V, D, W, DC, GE, L, I, P | Mental handicap in adults | 6 40-min sessions (6 wks) | Type & frequency of contact w/ mentally handicapped people; use of terms; knowledge of causes; attitudes |
| Brewer, 2004, England: Using Arts to Combat Stigma [68] | Post-test (150/NR) | NR (14-15) y | L, I, S, DC, A, D, V | NR | NR (3 days) | Perceptions of MH clients |
| Mound, 1993, Canada: *Beyond the Cuckoo’s Nest* [52] | Post-test (NR/NR) | NR (gr 11 & 12) | L, GE, D, DC, R | Auditory hallucinations, variety of disorders | 2 hr (1 day) | Pt feedback about experiences |
|  | | | | | | |
| **Author** = last name of first author, or acronym of organization having written the main report; **year** = year of publication of main report; **location** = country in which intervention was implemented; **intervention** = brief description of intervention or name of intervention if reported (names reported by authors are in italics); **MH** = mental health; **MI** = mental illness; **MHDs** = mental health difficulties; **pts** = participants; **grp** = group; **comparison groups** = type of intervention received by each grp of pts, including the grp receiving the target intervention & any control grps to which it was compared (**E** = intervention involving educational components only & no direct contact with individuals with MHDs; **C** = intervention whose sole or primary component was direct contact with an individual with MHDs or a MH professional; **E+C** = intervention involving both an educational & a direct contact component; **ctrl** = control grp; *****indicates an inappropriate control grp); **n-enrolled** = number of students who took part in study; **n-completed** = number of students who completed intervention & all related assessments; **†** = can’t tell from any retrieved documentation whether this number represents pts who enrolled or those who completed; **age of pts** = mean age (& range) of pts in years [e.g., 12.5 (11-14) y]; whenever possible, age data are broken down by comparison grps; if age data were not reported in any retrieved documentation, data on grade are included when available; **‡** = represents an average of mean ages that were reported separately for male & female pts; **intervention components** = types of activities in which student pts engaged during the intervention; interventions may have included more than one component of each type (**V** = video; **I** = written educational information (e.g., information leaflets, brochures, manuals); **S** = stories, cartoons, comic strips read by or to pts; **D** = discussion or question & answer sessions; **R** = role play, plays, skits; **DC** = direct contact with an individual with MHDs or a MH professional; **L** = lecture, verbal instruction; **A** = creation of artwork, music, posters etc.; **W** = creative writing (e.g., stories, essays, speeches); **GE** = grp exercises, activities, games; **IE** = exercises, activities, games etc. completed by pts individually **P** = pts presented program materials to others); **Q** = questionnaire or self-test completed by pts that were not part of any formal outcome assessment; **MH topics portrayed** = MH topics to which pts were exposed during the intervention; **length** = the amount of time that students spent participating in the intervention, not including time in between the intervention per se & any pre- &/or post-assessments; includes both the actual length of time (in hours or minutes) & the period over which the intervention occurred [e.g., 3 hours (3 days)]; **outcomes** = the measured dependent variables with respect to the final end-point in the evaluator’s analytic framework/model; **NR** = not reported in any retrieved report/documentation; **NBD** = data in report are not broken down (e.g., by grp); **CT** = can’t tell; **RCT** = randomized controlled trial; **M** = mean (average); **gr** = grade; **y** = year; **mo** = month(s); **wk** = week; **hr** = hour; **min** = minute(s); **vs** = versus; **w/** = with; **MR** = mental retardation; **OCD** = obsessive compulsive disorder; **ADHD** = attention deficit hyperactivity disorder | | | | | | |
